# Toxicity Profile of Chimeric Antigen Receptor T-Cell and Bispecific Antibody Therapies in Multiple Myeloma: Pathogenesis, Prevention and Management

**DOI:** 10.3390/curroncol30070467

**Published:** 2023-07-01

**Authors:** Mariam Markouli, Fauzia Ullah, Serhan Unlu, Najiullah Omar, Nerea Lopetegui-Lia, Marissa Duco, Faiz Anwer, Shahzad Raza, Danai Dima

**Affiliations:** 1 Department of Internal Medicine, Boston Medical Center, Boston University School of Medicine, Boston, MA 02118, USA; 2 Department of Translational Hematology and Oncology Research, Lerner Research Institute, Cleveland Clinic, Cleveland, OH 44195, USA; 3 Department of Hematology and Medical Oncology, Taussig Cancer Institute, Cleveland Clinic Foundation, Cleveland Clinic, Cleveland, OH 44195, USA; 4 Department of Pharmacy, Cleveland Clinic Foundation, Cleveland Clinic, Cleveland, OH 44195, USA

**Keywords:** CAR T-cell therapy, bispecific antibodies, cytokine release syndrome, neurotoxicity, hematotoxicity, infections

## Abstract

Multiple myeloma is the second-most common hematologic malignancy in adults worldwide. Despite ongoing advancement in therapeutic modalities, it remains an incurable disease with a 5-year survival rate of approximately 50%. The recent development and introduction of anti-BCMA immunotherapies into clinical practice, including chimeric antigen receptor T-cell (CAR-T) therapies and bispecific antibodies, has radically shifted the treatment paradigm. However, despite the promising potential of these therapies for broader application, frequent and significant adverse effects have been reported, both in short- and in long-term settings, requiring increasing awareness and vigilance in the treating team, close monitoring, and prompt interventions with a multidisciplinary approach. In this review, we will discuss the toxicities associated with CAR-T cell and bispecific antibody therapies, focusing on results from major clinical studies and real-world observations. In addition, we will emphasize on effective strategies for prevention, monitoring and management, and provide expert recommendations.

## 1. Introduction

Multiple myeloma (MM) is the second-most common hematologic malignancy in adults, characterized by the abnormal proliferation of monoclonal plasma cells (PCs) producing nonfunctional immunoglobulins [1]. In recent decades, significant therapeutic advancements have dramatically shifted the treatment paradigm, leading to increased survival and quality of life [1]. In spite of this significant progress, MM still remains an incurable cancer, emphasizing the need for new therapeutic strategies. 

Novel anti- B-cell maturation antigen (BCMA) therapies have recently emerged with impressive outcomes in heavily pretreated MM patients [2]. These mainly include chimeric antigen receptor (CAR) T-cell therapy and bispecific antibodies [3,4]. Currently, there are two CAR T-cell products and one bispecific antibody approved for clinical use [5,6]. Despite the high response rates and durable remissions, these therapies have been associated with a unique adverse event (AE) profile necessitating extra caution and close monitoring, as they can result in serious and potentially life-threatening complications. Major clinical trials have consistently shown that the most prominent AE include cytokine release syndrome (CRS), neurotoxicity, increased risk of infections and hematologic toxicities [7,8]. The degree and duration of toxicities vary among the different products and a significant percent of AE may be prolonged and challenging to manage with supportive measures. It is important, therefore, to consider the risks when choosing to use these novel modalities. In this review, we will focus on the toxicities associated with CAR-T cell therapies and bispecific antibodies currently used or tested in clinical practice, and discuss approaches for prevention and management.

## 2. Mechanism of Action

To better understand the underlying pathophysiology of the major AE of novel immunotherapies, it is important to first understand how these therapies work on a molecular level. Chimeric antigen receptors (CARs) are synthetic transmembrane proteins that are artificially constructed to selectively recognize specific antigenic epitopes on the surface of the tumor cells. They consist of the extracellular antigen recognition domain, and the intracellular segment, which includes the activation domain as well as costimulatory signaling endodomains that induce T-cell activation upon antigen binding [1,9]. CARs are placed on the surface of the patients’ physiologic T-cells, via a complicated ex vivo process, including T-cell collection, and transfer of the genes encoding the CAR constructs into the genome of T-cells via a vector, most commonly viral, ultimately resulting in the transcription and expression of CARs as surface receptors [10]. When ready, the CAR T-cells are administered as a single infusion after lymphodepleting chemotherapy that facilitates their proliferation and expansion by suppressing the innate immune system [9]. 

At present, the CAR T-cell products approved for clinical use in MM are idecabtagene vicleucel (ide-cel) and ciltacabtagene autoleucel (cilta-cel), both of which target the B-cell maturation antigen (BCMA) on the surface of PCs [11,12]. BCMA is primarily expressed by PCs with minimal or no expression by other tissues or other types of hematopoeitic cells. The interaction of CARs with their target on PCs leads to the activation of the CAR T-cells, which triggers a robust cytokine production from the CAR T and other endogenous immune cells, resulting in a massive immune response against the tumor [9].

Bispecific antibodies (bsAbs) are engineered hybrid molecules with dual binding capacity. They simultaneously bind to both a target epitope on the surface of the malignant PCs and on endogenous effector cells (usually T-cells, less frequently natural-killer [NK] cells), forming an immunologic bridge leading to the destruction of the tumor cells by the “activated” immune effector cell [13]. While several bsAbs are currently being tested targeting different antigenic epitopes, to date, only Teclistamab (TEC) has been granted approval for clinical use [5]. TEC binds to BCMA on the surface of PCs and CD3 receptor of the physiologic T-cells. Similar to CAR T-cells, the dual binding of TEC results in the activation of the endogenous immune cells triggering cytokine release surge [5]. Apart from BCMA, other molecular targets on PCs currently assessed include the GPRC5D and FcRH5 [14]. 

Both CAR T-cell therapies and bsAbs have been associated with a unique AE profile which can be serious, and potentially life-threatening, if not promptly addressed and treated. For this reason, it is a common practice for patients to be admitted to hospital for CAR T-cell infusion or bsAbs initiation with step-up dosing, for close monitoring and supportive care for a minimum of 9–12 days. The duration of hospitalization may be prolonged if serious complications arise [7,8]. 

## 3. Cytokine Release Syndrome

### 3.1. Pathogenesis

**Figure 1:**
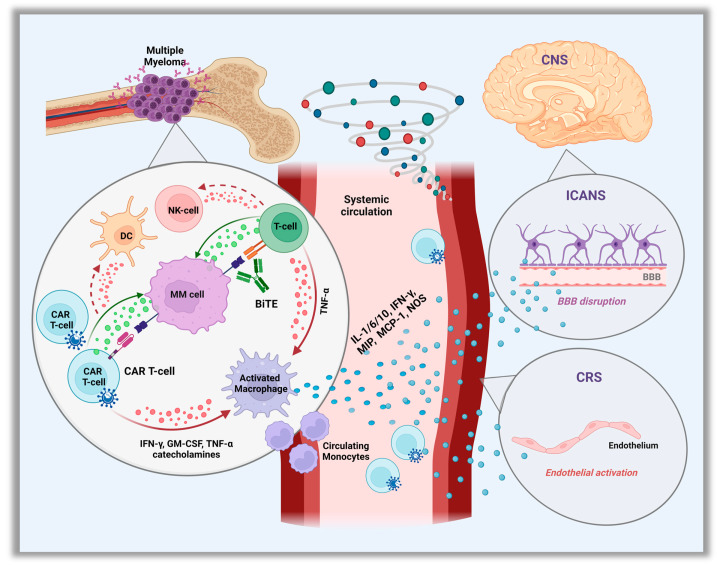
Pathophysiology of Cytokine Release Syndrome (CRS) and Immune Cell–associated Neurologic Syndrome. (ICANS). Abbreviation: CAR, chimeric antigen receptor; IFN-γ, interferon-γ; TNF, tumor necrosis factor; NOS, nitric oxide; GM-CSF, granulocyte–macrophage colony-stimulating factor; NK, natural killer; DC, dendritic cells; MIP, macrophage inflammatory protein; MCP-1, monocyte chemoattractant protein-1; CNS, central nervous system.

Cytokine release syndrome is a sequela of the overactivation of the immune response after administration of CAR T-cells or bsAbs [15], and constitutes the most common non-hematologic and well-described early onset toxicity of these therapies. However, it may vary between the different CAR T-cell products and bsAbs types. The interaction of the CARs with their target on PCs leads to the activation and local expansion of the CAR T-cells which triggers an in-situ cytokine hypersecretion such as interferon gamma (IFN-γ) or tumor necrosis factor α (TNF-α). These further activate local innate immune (monocytes/macrophages, T, B and NK cells) and non-immune (endothelial, dendritic cells) cells to produce a wide variety of cytokines, which play a central role in effector cell coordination [16,17]. As CAR T-cells continue to expand in the peripheral circulation, a massive cytokine storm occurs leading to a strong systemic inflammatory response (Figure 1). 

More than 100 cytokines, chemokines and other proinflammatory mediators are involved in the pathogenesis of CRS, with IL-6 being the major cytokine-driver [18]. IL-6 is predominantly produced by the endogenous macrophages/monocytes and also endothelial cells, and it is involved in several physiologic functions, including regulation of chemokine/cytokine secretion, and recruitment of immune cells during inflammation, angioge-nesis, and B-cell differentiation. Another important mediator is the IL-1 cytokine, also produced by macrophages/monocytes, and similar to IL-6, it has inflammation regulatory properties including amplification of IL-6 secretion [19,20]. Other contributing cytokines are the granulocyte-macrophage colony-stimulating factor (GM-CSF) and monocyte chemoattractant protein-1 (MCP-1), which, along with TNF-α and IFN-γ, play a role in migration and activation of macrophages and monocytes to produce IL-6 [16,17]. Apart from IL-1 and IL-6, macrophages also produce nitric oxide and catecholamines to further enhance the hyper-inflammatory immune response [16,21]. Given the central role of activated macrophages in the pro-inflammatory cytokine cascade production, secondary hemophagocytic lymphohistiocytosis (HLH) can potentially occur and has already been described in rare cases after the administration of CAR T-cell therapy [22].

Less is known regarding the exact mechanism of CRS associated with bsAbs. It appears that similar to CAR T-cells, the simultaneous binding of bsAbs to their antigenic targets on plasma and effector cells, induces the local release of cytokines such as IFN-γ or TNF-α, which further activate immune and non-immune cells to produce a mixture of proinflammatory cytokines [23]. Again, macrophages/monocytes are the major source of IL-6 production which regulates the cytokine storm. Interestingly, CRS has only been observed after the initial step-up dosing period, and rarely with subsequent full doses [24].

### 3.2. Presentation

CRS is characterized by a constellation of symptoms, most commonly fever, chills/rigors, fatigue, and anorexia. These symptoms can persist for several days, and progress to severe CRS, with hypotension, hypoxia, and organ dysfunction [19]. Organ dysfunction may be secondary to hypotension/hypoxia or from direct cytokine release effect, and includes arrhythmias, cardiac and renal failure, acute liver injury, and coagulopathy [20]. Despite the severity, organ impairment is reversible if CRS is recognized and treated promptly. It has been observed that bsAbs-induced CRS occurs earlier compared to CAR T-induced CRS, within a few hours of administration, whereas CAR T-induced CRS is usually seen a few days after infusion, when the cytokine release usually peaks [25]. 

The diagnosis of CRS may be challenging, due to the nonspecific nature of clinical symptoms and the lack of definite diagnostic testing. All patients require an extensive work-up to rule out infection, which includes routine labs, blood/urine cultures, and a chest X-ray at the minimum [26]. Inflammatory markers, such as ferritin, C-reactive protein (CRP), and erythrocyte sedimentation rate are usually elevated in patients with CRS, but again, are non-specific in diagnosing CRS [26]. IL-2, IL-6 and IFN-γ may also be elevated, however, these are not routinely measured in clinical practice. At present, the diagnosis of CRS is mostly clinical, prompting treatment with IL-6 inhibition (tocilizumab or siltuximab; see management section below). Emphasis, therefore, should be given in identifying more reliable biomarkers that will not only allow for an accurate diagnosis of CRS, but could also precisely predict development of severe CRS [27]. 

Clinical symptomatology of CRS appears to be milder in bsAbs than CAR T, with rare grade 3 and lack of grade 4 events. This is most likely due to the premedication with steroids given prior to bsAb administration and the step-up dosing strategy of bsAb initiation. In addition, there is limited ability of the native T-cells to cause extensive cytokine production compared to the large dose of artificial T-cells given with CAR T infusion. This was reflected in a recent meta-analysis, which reported higher overall and grade ≥3 CRS events, and longer CRS duration with anti-BCMA CAR T-cell therapies compared to bsAbs [28]. Interestingly, the proportion of CRS grade ≥3 was higher with bsAbs administered intravenously (IV) versus subcutaneously (SC), suggesting that the SC administration may allow higher doses, while reducing the incidence of high-grade CRS [15]. 

### 3.3. CAR T-Cell Therapy

Ide-cel was the first CAR T-cell product officially approved for heavily pretreated MM patients. The phase I CRB-401 study (n = 33) reported a CRS rate of 76% (70% grade 1–2, 6% grade 2–4) [29]. Median time from infusion to CRS onset was 2 days and the median duration was 5 days. CRS did correlate with CAR T-cell dose; higher doses led to higher CRS incidence. Interestingly, the incidence of CRS was associated with higher baseline ferritin levels, and involved free light chains and serum BCMA, all of which possibly indicate higher tumor burden. CAR T-cell expansion was higher in patients who experienced CRS compared with those who did not [29]. The following phase II KarMMa trial assessed ide-cel in patients with a median of six prior therapies [30]. The CRS rate reached 84%; however, events were mostly grade 1–2, with only 6% of patients experiencing grade 3–4 CRS. Median time from infusion to CRS onset and median duration of CRS were 1 and 5 days, respectively. Approximately half of the patients who developed CRS received tocilizumab (toci), and 15% glucocorticoids (GCs) [30]. No deaths were attributed to CRS complications. [30]. The recent KarMMa-3 phase III study compared ide-cel to standard regimens in 386 patients who had received 2–4 prior lines of therapy [12]. CRS occurred in 88% of the 225 patients who were randomized to the ide-cel arm, with only 5% developing an event of grade ≥3. For the two patients who developed grade 5 events, death occurred from organ failure and candida sepsis [12].

The Myeloma CAR T Consortium reported real-world efficacy and safety data of ide-cel in 159 heavily pretreated patients, 75% of whom did not meet the eligibility criteria for the KarMMa trial [31]. CRS of any grade, grade ≥2 and ≥3 occurred in 82%, 20%, and 3% of patients, respectively. Of them, 71% received toci, 5% anakinra, and 26% GCs for CRS, or neurotoxicity, or both; 8% were transferred to an intensive care unit (ICU). Three patients died from CRS grade 5, one of whom developed HLH. Univariable analysis showed that patients with an Eastern Cooperative Oncology Group (ECOG) performance status of ≥2, higher revised international staging system, and a bone marrow PC percentage of >50% at core biopsy prior to ide-cel, were more likely to develop grade ≥3 CRS [31].

The CARTITUDE-1 phase I/II study assessed the efficacy of cilta-cel in 97 patients with a median of 6 prior therapies [11]. CRS occurred in 95% of the cohort (51% grade 1, 39% grade 2, 4% grade 3–5). Median time to CRS onset was 7 days; in 89% of the cohort, CRS occurred after day +3. Median duration of CRS was 4 days. Of those who experienced CRS, 69% received toci, 22% GCs and 19% anakinra, with CRS ultimately resolving in 99% of cases. One patient with grade 5 CRS developed HLH and died on day +99. Two-year follow up analysis did not report any new events [32].

Other anti-BCMA CAR T-cell products are currently under rigorous evaluation, including Bb21217, which uses the same CAR molecule as ide-cel, but adds bb007, a PI3K inhibitor, to enrich the product in memory-like T-cells. The phase I CRB-402 study evaluated bb21217 in 72 patients, and reported a CRS rate of 75%, predominately grade 1–2. [33]. The CAR T-ddBCMA product, utilizing a novel synthetic binding domain, the d-Domain, yielded a CRS rate of 100% in a phase I study. The phase I EVOLVE trial evaluated orvacabtagene autoleucel (orva-cel) in 62 patients. While the overall CRS rate was not reported, grade ≥3 events were seen in 3% of patients [34]. Investigators gave prophylactic anakinra to 14 patients prior to the recommended orva-cel dose, and found it effective in reducing the incidence of grade ≥2 CRS, without impacting CAR T-cell expansion [35]. The LUMMICAR-2 phase Ib/II trial evaluated zevorcabtagene autoleucel in 14 patients, and reported CRS rate of 86%, with no grade ≥3 events [36]. G protein-coupled receptor, class C group 5 member D (GPRC5D) CAR T-cells have also been studied in early phase trials [37]. A phase II study of 33 patients reported a CRS rate of 76% (all grade 1–2), with median onset of 7 days; all events quickly resolved with toci or GCs [38].

Besides autologous CAR T-cell therapies, allogeneic CAR T-cell products have also been developed and assessed [39]. Allogeneic CAR T-cells are generated from T-cells of healthy donors and their manufacture process is faster than autologous products [39]. The UNIVERSAL phase I trial is evaluating the ALLO-715, a first-in-class allogenic anti-BCMA CAR T-cell compound, in 43 patients. Interim analysis reported CRS in 56% of participants, with only one patient experiencing grade ≥ 3 event [40].

### 3.4. Bispecific Antibodies

TEC is the only approved bsAb for clinical use in RRMM [41]. The phase I MajesTEC-1 trial (n = 157) reported an overall CRS rate of 57% and 70% in the recommended phase II dose group. All events were grade 1–2. Median time to onset (relative to the most recent dose) was 1 day with the IV dosing and 2 days with the SC dosing; median duration was 1 and 2 days, respectively. CRS resolved in all patients, of whom 24% required toci and 14.6% GCs [42]. The subsequent phase I/II trial reported that 72% of 165 patients developed CRS, which mostly occurred after step-up doses 1 (42%) and 2 (35%), or after the initial full-treatment dose (24%) [5]. Most events were grade 1–2 (71% of patients) and completely resolved; only one grade 3 event was reported in a patient with pneumonia and resolved 2 days later. Median time to onset (after the most recent dose) and median duration were both 2 days [5]. TEC was continued in all patients with CRS without interruption. Supportive measures were provided to 67% of patients, including toci (36%), GCs (8.5%) and a single vasopressor (1%).

Talquetamab (TALQ) is another bsAbs, which targets the GPRC5D molecule on the surface of PCs, along with the CD3 of T-cells [43]. Despite not being officially approved for clinical use yet, it is currently available via an expanded access program. A recent phase I trial, reported that CRS occurred in 77% and 80% of the patients in the 405 μg and 800 μg SC dosing arms, respectively, with only one grade 3 event. For patients who received IV TALQ, the CRS rate was 50% with five (5%) patients developing grade 3 events [44]. CRS mainly occurred with step-up doses and the first full dose. Median time to CRS onset (after the most recent dose) and median duration were 2 days, respectively, for both SC arms. Recurrent CRS (primarily during step-up and cycle 1) was noted in 30% of patients receiving the 405-μg SC dose and 27% receiving the 800-μg SC dose. Supportive treatment included toci (63% in the 405-μg dose level, 54% in the 800-μg dose level), mostly one dose, and GCs (3% in the 405-μg dose level, 7% in the 800-μg dose level) [44].

### 3.5. Prevention & Management

Pre-medications including acetaminophen, and diphenhydramine are administered one hour prior to CAR T-cell infusion and bsAb to prevent the incidence of CRS. For bsAb, in addition to the above, dexamethasone 16 mg oral or IV is also given as part of the pre-medication regimen [45]. Moreover, initiation of bsAb using a step-up dosing approach is another a strategy aiming to reduce the incidence of CRS. 

Preemptive use of toci has been associated with low CRS incidence and severity in patients with non-Hodgkin’s lymphoma treated with anti-CD19 CAR T-cell therapy [46,47,48]. In MM, prophylactic administration of toci and anakinra have been assessed prior to the first dose of bsAb or CAR T-cell therapy, respectively, with encouraging efficacy in reducing CRS. However, data are limited (deriving from studies with a small sample size), and are not strong enough to support the widespread use of prophylactic anti-cytokine agents [35]. Effort should be made to further evaluate prophylactic strategies in large cohorts of patients aimed at preventing CRS. It is equally important to identify predictive markers of severe CRS, where the application of prophylactic strategies would yield the highest benefit.

Management is based on the severity of CRS, with most low-grade events treated with supportive care (analgesics, antipyretics), intravenous fluids, and toci [19,28]. In cases of severe CRS and/or CRS refractory to toci, GCs should also be administered to inhibit the inflammatory cytokine production [28]. However, GCs should be used with caution, at the lowest possible dose and for the shortest duration, as it has been previously reported that steroids were associated with a negative impact on CAR T-cell expansion and efficacy [49], and may additionally affect the therapeutic activity of bsAbs [50]. Furthermore, in cases of persistent and/or high-grade CRS despite toci, other anti-cytokine agents may be utilized including siltuximab (IL-6 inhibitor) or anakinra (IL-2 inhibitor). If responses remain poor then tumor necrosis factor (TNF) inhibitors (infliximab, adalimumab, etanercept), JAK-2 inhibitors (ruxolitinib), or other T-cell suppressing therapies such as antithymocyte globulin (ATG), cyclophosphamide (Cy), or cyclosporine may be trialed.

As mentioned above, given that fever is the most frequent symptom of CRS, infectious work up should be always pursued in these patients, and antibiotic initiation should be strongly considered due to the profound accompanying immunosuppression. A detailed grading and management overview of CRS is summarized in Table 1 [51].

## 4. Neurotoxicity

### 4.1. Pathogenesis 

Neurotoxicity (NT) is another common AE observed, and its association with the CAR T-cell therapy is well known. In the KarMMa and CARTITUDE trials, most episodes of NT occurred in proximity to CRS and the most commonly reported symptoms were encephalopathy and confusion [29,30]. Despite this association, NT appears to be a pathophysiologically distinct phenomenon [52]. Most acute and early-onset NT is believed to be caused by immune effector cell–associated NT syndrome (ICANS), which is a well-described entity with CD-19 CAR T-cell therapies for non-Hodgkin’s lymphoma or B-cell acute lymphoblastic leukemia [53,54]. However, the exact pathogenesis in the setting of anti-BCMA CAR T is less clear, though, it is thought to be an off-target toxicity. Speculated mechanisms include: cytokine-induced endothelial activation with blood–brain barrier disruption, and thus, direct central nervous system (CNS) exposure to blood-circulating cytokines. Microglia are then likely activated by the series of cytokines migrating to the cerebrospinal fluid (CSF), thereby triggering secondary cytokine production and local inflammation. Additional mechanisms include activation of myeloid cells and by-standing monocytes, as well as CNS infiltration by CAR T cells [55]. 

Interestingly, there is some evidence suggesting that BCMA expression in not only limited to the hematopoietic system, but it also occurs in the nervous system. Studies in murine models suggested that BCMA may have a role in hippocampal development [56]. In addition, data from the Allen atlas of the human brain indicated expression of the BCMA gene in several locations of the CNS, with most prominent being the subcortical areas, including the caudate nucleus and putamen of developing embryo and adult brains. These findings imply that NT may be an on-target off tumor toxicity against BCMA of nervous tissue [57].

### 4.2. Presentation

The onset and duration of NT is highly variable [54]. Early NT or ICANS is usually mild and typical symptoms include word-finding difficulties, confusion, delirium, transient aphasia, bradyphrenia, agitation, hallucination, tremor, dizziness, vertigo, encephalopathy, and polyneuropathy/nerve palsy. However, more severe symptoms that need immediate attention and intervention including cerebral edema, obtundation, and seizures, have also been described [54,55]. ICANS is often preceded by CRS, but sometimes both entities can occur at the same time. Aside from CRS, other risk factors for ICANS development include preexisting neurologic comorbidities, high tumor burden, and intensity of lymphodepleting chemotherapy [58]. The major agents utilized in ICANS management are GCs (discussed below in the management section).

Apart from ICANS, non-ICANS late NT can occur, manifesting as movement and neurocognitive treatment-emergent AEs (MNTs). This usually develops after the recovery period from CRS and/or ICANS and has a longer and usually irreversible nature [59]. Similar to ICANS, these phenomena are also clinically heterogeneous, and may mimic parkinsonism, with movement (tremor, bradykinesia, shuffling gait, apraxia, rigidity, micrographia), cognitive, and personality changes (memory loss, flat affect, etc.) [60]. 

### 4.3. CAR T-Cell Therapy

When looking at landmark trials, there is a lack of a uniform definition of NT. The CARTITUDE-1 trial reported ICANS and non-ICANS NT separately, while ide-cel trials, including KarMMa, did not. In CRB-401, NT occurred in 42% of patients, with 39% being grade 1–2 [33]. In KarMMa, 18% of patients developed NT with 3% being grade 3 [30]. No grade 4–5 events were reported. Median time to any NT was 2 days and median duration was 3 days. The onset of NT was during CRS in the majority of patients. The most common manifestations were encephalopathy (20%), tremor (9%), aphasia (7%), and delirium (6%) [30,61]. Ide-cel caused NT in 15% of patients in the KarMMa-3 study (grade ≥3 in 3% of patients); median time to onset was 3 days, and GCs were administered to 7% of patients [12]. Notably, cerebral edema (grade 4), myelitis (grade 3), and parkinsonism (grade 3) were reported in other studies of ide-cel [62].

Real-world data from Myeloma CAR T consortium indicated an 18% rate of NT, with 3% of patients suffering from grade 3 and 3% from grade 4 events. Median time to maximum severity was 3 days. As mentioned above, 71%, 5%, and 26% of patients received toci, anakinra, and GCs, respectively, for CRS and/or NT. One patient developed progressive ascending weakness resulting in death. ECOG performance status of ≥2, elevated baseline ferritin and B2 microglobulin, use of bridging chemotherapy, and higher CAR T-cell dose (≥400 × 10^6^) were associated with grade ≥2 NT [31].

In CARTITUDE-1, general NT was seen in 21% of patients, with ICANS occurring in 17% (15% grade 1–2, 2% grade 3–4), and non-ICANS NT in 12% (3% grade 2, 7% grade 3, 1% grade 4, 1% grade 5); while 8% of patients experienced both types of NT [11]. Median onset of ICANS was 8 days after the infusion and median duration was 4 days. All patients with ICANS also experienced CRS. The most frequent manifestations included encephalopathy (23%), aphasia (8%), and headache (6%). GCs were administered in 9% of patients, toci in 4% and anakinra in 3%, with ICANS ultimately resolving in all cases. Median time to late-onset NT was 27 days, with complete resolution in 50% of cases (median time to recovery was 75 days) [11]. Presentations were heterogenous including, peripheral neuropathy, cranial nerve palsies (resolved with GCs), and parkinsonism/MNTs (median onset 43 days) [63], which did not respond to various treatments with GCs, chemotherapy, or dopaminergic agents; only one patient had partial resolution without any specific therapy. Patients who suffered from MNTs were more likely to have at least two of the following: high tumor burden, prior grade ≥ 2 CRS, previous ICANS, or high CAR T-cell expansion/persistence. Among the six non-ICANS patients whose symptom did not resolve, one died from grade 5 parkinsonism, four died from other causes but NT had not resolved at time of death (2/4 had parkinsonism), and the last was alive with ongoing NT at the data cutoff [29]. Other delayed NTs of cilta-cel in different trials include: facial paralysis, Guillain-Barré syndrome, and immune-mediated myelitis [64]. 

For non-approved anti-BCMA products, 17% of patients who received ddBCMA experienced NT (50% grade 1–2, 50% grade 4); for grade 1–2 events, median time to onset was 2 days and median duration was 12 days, whereas for grade 4 events, these were 6 and 14 days, respectively [65]. At the last update of the EVOLVE study, 3% of patients developed grade ≥ 3 NT. None of the patients in the LUMMICAR-2 study experienced grade ≥ 3 NT. In the UNIVERSAL trial of allogeneic CAR T-cells, only one patient had NT. Low rates of NT (9%) were also noted with anti-GPRC5D CAR T-cells [59]. An incidence of grade 3 cerebellar toxicity was described, however, the cause was unclear [37].

### 4.4. Bispecific Antibodies

NT is less well-described in association with bsAbs. The MajesTEC-1 trial reported overall NT in 14.5% of patients who received TEC, including ICANS in five patients (3%). Most NT events were grade 1–2 (ICANS was 100% grade 1–2), except for two grade 4 events, presenting as a seizure in the setting of bacterial meningitis [5], and fatal Guillain–Barré syndrome. Headache was the most common NT (8.5%). Other symptoms included motor dysfunction, sensory neuropathy, tremor, lethargy, and encephalopathy [66]. Most ICANS events followed step up doses and the initial full dose, with median time to onset (after the most recent dose) and median duration being 4 and 3 days, respectively [66]. The most common clinical presentations of ICANS were confusion and dysgraphia. All ICANS events ultimately resolved without dose reduction or interruption. No patient discontinued TEC due to NTs. Supportive care was utilized in 8.5% of patients, and included toci (2%), GCs (2%), levetiracetam (1.2%), and gabapentin (0.6%) [5]. 

In the MonumenTAL-1 trial, treatment-related NTs were reported in 10% and 5% of patients in the 405-μg and 800-μg SC dosing arms of TALQ, respectively [44]. These events were all of grade 1–2 and quickly resolved. Single events of encephalopathy, confusion, aphasia, and anosmia were additionally reported, mostly during step-up doses and first full dose. Of the entire cohort, including patients who received IV TALQ, 3% experienced grade 3 NT events [44]. 

### 4.5. Prevention & Management

The major agents used for ICANS management are GCs; type, dosing, frequency, and duration are based on the grade (Table 2) [67]. For grade 1–2 events, dexamethasone 10 mg every 12–24 h should be started. If ICANS persists or events are grade > 2, then higher doses of dexamethasone in shorter time intervals, or methylprednisolone should be given instead. If ICANS is refractory to GCs, other agents can be added such as anakinra or lymphotoxic agents (e.g., Cy). Because ICANS typically develops a few days after CRS, effective management of CRS with IL-6 inhibitors is of critical importance to prevent ICANS. Once ICANS occurs, treatment with IL-6 inhibitors is much less effective, however, in cases of coexistent CRS, they should be given. For patients with ICANS grade ≥ 3, transfer to an ICU might be necessary for continuous monitoring. Severe ICANS may require mechanical ventilation for airway protection.

It is important to consult a neurology specialist early in the course to assist with work-up. Initial assessment should include head computed tomography to rule out intracranial bleeding, which may occur in the setting of cytopenias. Further evaluation with brain MRI, electroencephalogram and lumbar puncture should be pursued in selected cases of refractory ICANS despite GCs treatment. Patients with cerebral edema and increased intracranial pressure should be started on hyperosmolar therapy and continuously monitored in a neurocritical ICU. 

For late non-ICANS NT, given its rare nature, optimal management is not yet determined and, in most cases, the approach is physician dependent. Again, GCs are first-line agents. Supportive measures and individualized management are also important. For example, in patients with parkinsonism, drugs used in Parkinson’s disease can be theoretically utilized, including levodopa, dopamine agonists, inhibitors of enzymes that inactivate dopamine, anticholinergic drugs, and amantadine; however, efficacy is not very encouraging based on the available limited data [60].

## 5. Infections

### 5.1. Pathogenesis 

Patients who receive anti-BCMA therapies are at an elevated risk for serious infections due to the profound and prolonged immunosuppression. Due to the abundant expression of BMCA, GPRC5D and other target epitopes on healthy PCs, novel therapies can cause non-selective PC eradication, resulting in severe hypogammaglobulinemia [68,69]. In addition, prolonged cytopenias (especially neutropenia), T-cell exhaustion, and reduced bone marrow reserves from primary disease and pervious therapies are other elements contributing to the increased infection risk. Furthermore, severe CRS with massive releases of cytokines can cause a form of immune paralysis which further predisposes patients to infections [26]. Overall, there is a significant heterogeneity in infections reported by major clinical trials and retrospective analysis with regard to type of pathogen, location of infection, and severity, however, it is clear that there is an overall elevated risk for severe COVID-19 infection with a high mortality risk of up to 50% [70]. Deaths from neutropenic infections have also been reported.

### 5.2. CAR T-Cell Therapy

Ide-cel resulted in a 42% infection rate in the CRB-401 study, with only 6% being grade 3, but none being grade 4 [29]. In the KarMMa trial, 69% of participants developed infections with 22% being grade 3–4. Most infections were viral (27%), followed by bacterial (15%) and fungal (8%), while the rest were due to unspecified pathogens [30]. Grade 5 infections were seen in five patients: two had pneumonia, one bronchopulmonary aspergillosis, and one had cytomegalovirus (CMV) pneumonia associated with *P. jirovecii.* Infection incidence and the severity did not differ between the various doses. Antimicrobial agents, growth factors, and intravenous immunoglobulin (IVIG) were used in most patients. In KarMMa-3, 58% of patients in the ide-cel arm developed infection; of these, 24% were grade 3–4 and 4% were grade 5 [12]. Upper respiratory tract infections were the most common, seen in 12% of the ide-cel cohort, followed by pneumonia in 10%. The most common grade 5 AE was sepsis, which occurred in five (2%) patients, one of which was candida bacteremia [12].

Real-world data show similar rates of infection. The Myeloma CAR T Consortium reported an infection rate of 34% after ide-cel, with the majority being bacterial (20%), followed by viral (16%) and fungal (1%) [31]. Another retrospective study reported an early (until day 100 post infusion) infection rate of 54% post ide-cel, with 23% being grade ≥ 3. Infections that occurred between day 1–30 from infusion were mostly bacterial (68%) and severe (50%). Similarly, infections that occurred between day 31–100 post-infusion, were predominately bacterial (50%); however, only 13% were grade ≥3 [71]. Another single-center study of 55 patients reported a 53% infection rate after a median follow up of six months, most of which were viral (53%) followed by bacterial (40%). The majority (92%) were mild–moderate, while 68% involved the respiratory tract system. Notably, 50% of the reported infections occurred during the first 100 days after infusion [21].

In the CARTITUDE-1 trial, 58% of 97 participants developed infections, of which 20% were grade 3–4 [11]. The most frequent infection type was of upper respiratory tract (16%), whereas the most common grade 3–4 infection types were pneumonia (8%) and sepsis (4%) of unspecified pathogens. Overall, four patients experienced grade 5 infections: lung abscess (1), sepsis (2), and pneumonia (1). The ongoing CARTITUDE-4 trial has preliminarily reported an increased rate of fatal COVID-19 infections compared to standard regimens [63]. Other studies of cilta-cel have reported bronchopulmonary aspergillosis, mycotic aneurysm/cerebral aspergillosis, *P. jirovecii* pneumonia, and CMV colitis (with HSV-1 hepatitis) [63].

In the phase I study of CART-ddBCMA, no grade 3–4 infections were reported. The incidence of grade ≥ 3 infections in the EVOLVE study was 13%, and no correlation between infection incidence and dose was noted. No severe infections were reported with anti-GPRC5D CAR T-cell therapy during the neutropenic phase after a median follow-up period of 5.5 months [38]. Grade ≥ 3 infections occurred in 13% of patients in the UNIVERSAL trial of allo-CAR T, including two grade 5 events (fungal pneumonia, adenoviral hepatitis) [40]. 

### 5.3. Bispecific Antibodies

In the MajesTEC-1 trial, TEC led to an infection rate of 76%, with 45% being grade 3–4 events [5]. Most infections were of the respiratory system followed by the urinary tract system. COVID-19 infection was seen in 18% of the cohort with 12% being grade 3–4, resulting in 12 deaths. Two patients discontinued TEC due to grade 3 adenoviral pneumonia and grade 4 progressive multifocal encephalopathy. Other serious grade 3–5 infections included pneumonia (13%), and *P. jirovecii* infection (4%) [5]. Reactivation of CMV, hepatitis B, varicella zoster, and herpes simplex virus have also been reported with TEC. 

The rate of infections was lower with TALQ. Infections were noted in 47% of patients who received the 405-μg dose (grade 3–4 infections in 7%) and 34% of those who received the 800-μg dose (grade 3–4 infections in 7%) [44]. Opportunistic infections occurred in a total of six patients, of whom two had received 405-μg of SC TALQ, two 800-μg of SC TALQ and the rest had received IV TALQ. These included adenovirus infection (2 patients), esophageal candidiasis (2 patients), disseminated varicella zoster (1 patient) and ophthalmic herpes (1 patient). No CMV reactivation occurred; however, COVID-19 infection occurred in 13% and 2% of patients who had received the 405-μg and 800-μg SC dose levels, respectively. No infection-related deaths were documented [44]. Overall, the infection profiles of SC and IV TALQ were similar. 

A retrospective study assessed the risk of infection in 62 patients who received anti-BCMA therapies, including bsAbs (n = 36) and CAR-T-cells (n = 26). After a median follow-up period of 9 months, the cumulative incidence of infection with bsAbs and CAR-T separately, were 25 and 5, with 41% and 23% of patients in the bsAbs and CAR T groups, experiencing at least one infection, respectively [69]. Infections were predominantly bacterial, and about 50% were grade ≥ 3, with two being grade 5 (COVID-19 and Pseudomonas bacteremia), both in patients who had achieved complete responses on bsAbs therapy. For the CAR T group, more infections occurred within the first 30 days, whereas in the bsAbs group, the median time to infection was 49 days from initiation. Authors concluded that the higher rate and severity of infections with anti-BCMA bsAbs could be possibly due to the uninterrupted nature of therapy, resulting in persistent B-cell aplasia and hypogammaglobulinemia [69]. Other retrospective analyses and metanalyses have also highlighted the infectious complications with bsAbs suggesting that baseline hypogammaglobulinemia, uninterrupted therapy, previous infection and elevated monoclonal proteins were associated with increased risk of infections [72,73]. Detailed AE profile including infections of clinically tested bsAbs are summarized in Table 3 [74,75,76,77,78,79,80,81,82,83,84,85,86,87,88,89,90,91].

### 5.4. Screening, Prevention and Management 

Given the perplexity and heterogeneity of infectious complications, close monitoring and preventive measures are critical. As a rule, the presence of any active, and/or uncontrolled infection, therapy should be postponed until the infection has completely resolved.

#### 5.4.1. Viral Infections

Prior to receiving CAR T-cell therapy or initiating bsAb therapy, all patients should be screened for viral infections, including CMV, hepatitis A (HAV), B (HBV) and C (HCV) virus, human immunodeficiency virus (HIV), and COVID-19, as these could reactivate, posing serious and potential life-threatening complications [21,51,69,91,92].

The safety of novel therapies in the setting of active, chronic, or previous HBV infection is unknown as these patients were excluded from clinical trials. Patients with low or undetectable grade HBV viremia and lymphoma were reported to be successfully treated with anti-CD19-CAR T-cell therapy in the past; however, the sample size was very small [93]. In these cases, decision regarding candidacy should be individualized. If a patient is deemed eligible, anti-viral therapy with entecavir is mandatory in carriers (HBsAg-positive, patients with detectable HBV DNA load) and patients with previous infection (HBsAg-negative but anti-HBc-positive) [94]. Entecavir (or alternatively tenofovir) prophylaxis should be maintained for at least 6–12 months post-CAR T-cell infusion, or as long as the patient is on bsAbs (sometimes longer based on individualized factors).

Likewise, patients with HCV infection should be considered for novel therapies on a case-by-case basis with the concomitant administration of antiviral treatment [95]. There are no data for patients with HIV, as these were excluded from all trials, apart from three reported cases where patients were successfully treated with anti-CD19-CAR T-cell therapy for lymphoma [96,97].

Prophylaxis with acyclovir or valacyclovir should be started in all patients who are seropositive for Herpes simplex virus/Varicella-zoster virus at the time of lymphodepletion until at least day +100 after therapy, or while the patient is on bsAbs. Seronegative patients do not require prophylaxis [51,98]. Prophylaxis or monitoring of CMV levels are not currently recommended in routine practice given the lack of supporting data. However, CMV levels should be checked in selected clinical scenarios where indolent CMV infection could be the cause of the patient’s signs or symptoms (such as cytopenias, fevers of unknown etiology, colitis, etc.).

Regarding COVID-19, immunization prior to therapy initiation and revaccination after CAR T-cell infusion is recommended. The pre-exposure COVID-19 prophylaxis monoclonal antibody, Evusheld, is also recommended for this patient population [99]. In the setting of acute infection patients should be treated with remdesivir and steroids. Infectious disease specialist should be consulted early to assist with management. Convalescent plasma and therapeutic IVIG may also be considered for active infection. 

#### 5.4.2. Fungal Infections

Serious fungal infections with *P. jirovecii*, aspergillus and candida have also been reported by major trials. Routine prophylaxis with fluconazole is controversial. Some institutions recommend initiation post-lymphodepletion for CAR T-cell therapy, and continuation at least until neutrophil recovery occurs or during bsAb therapy. However, other centers, including ours, do not recommend the initiation of empiric prophylaxis for all patients. Instead, this decision is based on individualized parameters and the clinical trajectory [98]. If toci, siltuximab, anakinra and/or high-dose steroid are administered; general recommendations suggest antifungal prophylaxis for at least one month after the last dose [98]. Furthermore, in cases of prolonged and severe neutropenia (ANC < 500/mm^3^), antifungal prophylaxis initiation is reasonable. 

Trimethoprim-sulfamethoxazole (first line) or inhaled pentamidine for *P. jirovecii* infection prophylaxis, is mandatory post-CAR T-cell infusion (start on day +30) or during BsAb therapy. For CAR T-cell recipients, prophylaxis is usually continued for 6 months or until CD4+ count is ≥200 cel/mcL but can be continued longer per the provider’s discretion. Alternative agents are dapsone, and atovaquone.

#### 5.4.3. Bacterial Infections

Bacterial infections, especially pneumonia, are among the most common infections in patients receiving CAR T-cell or bsAbs therapies. The use of antibacterial prophylaxis in mitigating the risk of serious bacterial infections in non-neutropenic patients is controversial and is not generally recommended [100]. In selected cases, if ANC falls below 500/mm^3^ for a prolonged period, pharmacologic prophylaxis should be started and given until counts recover. Levofloxacin is the most common antibiotic used for prophylaxis; other alternatives include amoxicillin/clavulanic acid and cefdinir. For patients with severe neutropenia, additional support with granulocyte colony-stimulating factor (G-CSF) should also be considered. G-CSF should not be used within 2 weeks of CAR T-cell infusion and during the step-up dosing of bsAb to mitigate the risk of CRS. Other prophylactic measures include IVIG administration and vaccinations which are mentioned below.

In patients with symptoms of potential bacterial infection such as fevers, infectious work up should be completed, and empiric treatment with broad spectrum antibiotics should be started, especially in cases of neutropenia. If no infectious organism is identified after 48 hours and the patient remains afebrile and hemodynamically stable, de-escalation or discontinuation of antibiotics can be considered.

#### 5.4.4. Vaccinations

Prior to lymphodepletion or bsAb initiation, immunization of patients and their families with the influenza and COVID-19 vaccinations is strongly recommended, though efficacy may not be optimal [51]. At therapy initiation and during the B-cell depletion phase, live or attenuated vaccines are contraindicated. However, after immune reconstitution and recovery of B-cell aplasia, all re-vaccinations should be repeated in patients who received CAR T-cell therapy (usually 3–6 months after the infusion), including influenza, COVID-19, and pneumococcal conjugated vaccines [51,101].

## 6. Hematologic Toxicities 

Hematologic toxicity (HT) with cytopenias is a common phenomenon post-CAR T-cell infusion or with bsAbs [102,103,104]. Grade 3–4 HT can occur early or late onset, starting less or more than 30 days post-infusion, as well as in the short-term, up to 60 days post-infusion, or prolonged, persisting beyond 90 days. Common risk factors for severe hematotoxicity include old age, intensity of lymphodepleting regimen prior to CAR T, high tumor burden, heavily pretreated disease, low baseline peripheral counts, and bone marrow reserve with hypoplastic picture on bone marrow biopsy, as well as a high CRS grade with increased inflammatory markers. However, the exact underlying contributing mechanisms are still unclear [7,102,105,106]. Severe hypogammaglobinemia is another challenge in patients receiving these novel drug classes.

### 6.1. CAR T-Cell Therapy

Hematologic AEs were the most common grade ≥ 3 events that occurred in the CRB-401 trial [29]. Overall, 85% of patients developed neutropenia (100% grade 3), 58% developed anemia (45% grade ≥ 3), and 58% developed thrombocytopenia (45% grade ≥ 3). Among patients with grade ≥ 3 cytopenias, 97% recovered to an ANC of ≥1000 cells/mm^3^ and 65% to a platelet count of ≥50,000 cells/mm^3^ by month 1, with a median time from infusion to ANC and platelet recovery being 1.3 and 2 weeks, respectively [29]. In KarMMa, grade 3–4 neutropenia was seen in 89% of patients, anemia in 60%, and thrombocytopenia in 52% [30]. Notably, four grade 3–4 bleeding AE were reported, including cerebral, gastrointestinal, conjunctival, and postprocedural hemorrhage. Among patients with prolonged (duration >1 month post infusion) neutropenia (41%) and thrombocytopenia (49%), the median time to count recovery to grade ≤2 was 1.9 and 2.1 months, respectively. Hypogammaglobulinemia was seen in 21% of patients, the majority of whom received IVIG [107]. Similarly, in KarMMa-3, the most common HT in the ide-cel arm was neutropenia (78%), anemia (66%) and thrombocytopenia (54%) [12]. For patients with prolonged grade 3–4 neutropenia or thrombocytopenia, the median time to recovery was 1.7 and 1.9 months, respectively.

A multi-center study of 47 patients, reported a 65% rate of grade ≥ 3 cytopenia at day +30 and 40% at day +90 [31,71]. G-CSF as given to 88% of patients, red blood cell transfusions to 63%, platelet transfusions to 42%, thrombopoietin receptor (TPO) agonist to 21%, IVIG to 13%, and CD34+ stem cell boost to 8%. On day +100, 19% and 13% of patients were on a TPO agonist and G-CSF, respectively. A larger study from the same group reported that ide-cel led to any-grade neutropenia in 97% of patients (≥3 grade in 88%), any-grade anemia in 95% (≥3 grade in 51%), and any-grade thrombocytopenia in 95% (≥3 grade in 68%) of the cohort. Grade ≥ 3 HT persisted for >30 days after the infusion in 60%, 38% and 59% of patients with neutropenia, anemia, or thrombocytopenia, respectively. G-CSF was administered to 74% of patients, TPO agonist to 15% and stem-cell boost to 5% [31,71]. Another study of 90 patients who received anti-BCMA CAR T reported any grade ≥ 3 ongoing cytopenia in approximately one third of patients four months after the infusion. Median time to recovery to grade 2 or lower cytopenia was approximately 2 months [105].

Likewise, HT was the most common AE in the CARTITUDE-1, where 95% of patients experienced grade 3–4 neutropenia, 68% grade 3–4 anemia, and 60% grade 3–4 thrombocytopenia. The percentage of patients with grade 3–4 cytopenia events that recovered to grade ≤2 by day +30 was 70% for neutropenia, 88% for lymphopenia, and 59% for thrombocytopenia. Of patients with grade 3–4 HT, 90% and 58% had recovered their neutropenia and thrombocytopenia, respectively by day +60 [11]. HT of non-approved products has also been high in early phase trials. Studies on anti-GPRC5D CAR T-cells reported HT as the most frequent grade ≥ 3 AE including neutropenia (100%, median duration 14 days), anemia (52%, median duration 24 days), and thrombocytopenia (45%, median duration 29 days) [38].

### 6.2. Bispecific Antibodies

The most common AE of TEC in the MajesTEC-1 trial was HT, with neutropenia (71%), anemia (52%), and thrombocytopenia (40%) [5]. Most of events were grade 3–4: neutropenia 64%, anemia 37% and thrombocytopenia 21%. Among patients who developed neutropenia, 78% received G-CSF. Hypogammaglobulinemia (IgG < 500 mg/dL) occurred in 74.5% of patients, of whom approximately half received IVIG [5]. Likewise, hematologic AEs were the most common grade 3–4 events of TALQ in the MonumenTAL-1 trial. Cytopenias were reversible and mostly limited to patients who had received step-up doses and early full doses through cycle 2. Hypogammaglobulinemia occurred in 87% of patients who had received the 405-μg dose and in 71% of those who had received the 800-μg dose [44]. HT rates of other bsAbs are summarized in Table 3. A retrospective study of 42 patients treated in early phase I and II clinical trials of bsAbs reported that after a median follow-up of 9.5 months, 28% of the cohort had hypogammaglobulinemia, with 50% and 60% of patients having low IgA and IgM levels, respectively [72]. In another study, among the 37 patients who were treated with anti-BCMA bsAbs, 70% developed severe hypogammaglobulinemia with IgG levels < 200 mg/dL [68].

### 6.3. Prevention & Management

It is important to perform a baseline patient evaluation, to rule out other underlying etiologies that could increase the risk for inordinate HT. Hospitalization for CAR T-cell infusion is currently recommended for transfusional support, in addition to CRS/ICANS monitoring. Daily inpatient laboratory evaluation should include a complete blood count with differential, comprehensive metabolic panel, coagulation studies, CRP, lactate dehydrogenase, uric acid, and ferritin, however some patients with electrolyte wasting, coagulopathies or need for transfusion may need more frequent lab work [108].

More recently, there has been an increasing effort to shift this intervention in the outpatient setting. However, more data are needed with regard to safety and feasibility [109]. In an attempt to prevent severe cytopenias, it is crucial to provide immediate early supportive intervention in patients with high-risk baseline characteristics, as well as possibly modify lymphodepleting protocols that may predispose patients to higher-grade HT, such as replacing cyclophosphamide with bendamustine. However, the role of lymphodepleting therapy needs to be further studied in this context [110]. Reasonable application of corticosteroids and toci for CRS is also important, as persistent CRS appears to be a common risk factor for severe early cytopenia [111].

If severe hematological AEs occur, management includes a series of therapeutic measures to prevent bleeding and/or serious or opportunistic infections. In cases of prolonged thrombocytopenia, in addition to transfusional support, TPO initiation (such as romiplostim, eltrombopag) should be strongly considered [112]. The use of prophylactic G-CSF in the setting of severe neutropenia was discussed above. For patients whose counts do not recover in a timely manner post-CAR T-cell therapy, stem cell boosts are an option [102]. 

Prophylactic administration of IVIG is recommended in patients with hypogammaglobulinemia (IgG < 500 mg/dL) to mitigate risk of infection. IVIG supplementation should be performed every 4 weeks. In CAR T-cell recipients, it should be started around day +30 and continued until IgG > 400 mg/dL or up to 1 year. For patients on bsAbs, IVIG can be started at cycle 2, and should be continued throughout the duration of bsAb therapy. If stable recovery of IgG > 400 mg/dL is achieved, cessation of IVIG replacement is a reasonable option, especially in the absence of recurrent infections. Moreover, IVIG should be used therapeutically to help control moderate to severe acute infections in patients with low IgG levels or patients with IgG levels in normal range, but recurrent infections despite prophylactic antibiotics [108,113].

## 7. Other Toxicities 

### 7.1. Hemophagocytic Lymphohistiocytosis

HLH is a rare and life-threatening complication of CAR T-cell therapy caused by the excessive activation of the immune system and massive cytokine release, with subsequent tissue infiltration by lymphohistiocytic cells leading to multiorgan failure [114]. CRS usually precedes HLH by a few days; however, in very rare cases, HLH can develop weeks later after resolution of CRS. Patients usually present with high fevers, pancytopenia, and elevated inflammatory markers, especially ferritin. HLH is usually refractory to IL-6 inhibitors requiring additional immunosuppression with other agents (anakinra). However, responses are generally poor with a high mortality rate. 

### 7.2. Skin & Nail-Related Adverse Events

CAR T-cell therapies and bsAbs (such as TALQ) targeting the GPRC5D can affect tissues containing keratin due to the expression of GPRC5D by these tissues, resulting in off-target AEs. Symptoms include rash (mainly desquamation of hands and feet), nail changes, dysgeusia, loss of appetite, and dry mouth. Management of these AEs includes the topical application of moisturizing lotion, ammonium lactate lotion, and topical steroids. Adequate hydration is important and should always be encouraged [44,115]. 

## 8. Conclusions

Despite the tremendous progress in the therapeutic landscape of MM with the recent introduction of novel anti-BCMA therapies, new challenges continue to arise due to the unique toxicity profile of these modalities. Clinical trials and real-world observations have thus far provided valuable insight with regard to timing, frequency, duration, and outcomes of AE, which can often lead to increasing morbidity and mortality, if left untreated. At present, due to the complexity of administration and high rates of AE, these therapies are only administered in tertiary centers that have the appropriate expertise and staffing to provide the necessary care and close follow-up monitoring. This results in limiting accessibility to only a small proportion of patients. Efforts are currently being made to shift the direction to the outpatient setting, which could perhaps broaden accessibility; however, early-onset associated toxicities still remain the biggest challenge for this transition. Apart from the physical risks, given the prolonged nature of these toxicities and need for specialized management, costs and financial burdens are also very important aspects for patients and their families, and should be discussed prior to proceeding with these therapies. 

The management of novel therapy-related toxicities has significantly evolved; however, late-onset toxicities, neurotoxicity in particular, are not yet well-characterized, resulting in suboptimal treatment and significant impact on quality of life. Further research is needed to elucidate the underlying mechanisms mediating these AE, with additional hope to shed light on important biological predictors of toxicity. To date, there is no global guideline on the evaluation and management of CAR T-cell therapy or bsAb-related toxicities, posing an additional challenge. The establishment of optimized guidelines is therefore imperative to avoid confusion, standardize clinical trials, and proceed toward improving care of these patients. 

## Figures and Tables

**Table 1:** CRS Grading and Management per the American Society of Blood and Marrow Transplantation Consensus.

	Grade 1	Grade 2	Grade 3	Grade 4	Grade 5
**DEFINITION**					
**Fever, Temp ≥ 38 °C**	YES	YES	YES	YES	Death
Plus					
**Hypotension**	NO	YES, but not requiring vasopressors	YES, 1 vasopressor, with or without vasopressin	YES, ≥1 vasopressors, excluding vasopressin	
And/or					
**Hypoxia**	NO	YES, low-flow nasal canula or blow by	YES, HFNC, facemask, non-rebreather or Venturi mask	YES, positive pressure (CPAP, BiPAP, intubation with mechanical ventilation	
**MANAGEMENT**					
	Supportive care IV fluids Infectious work-up Consider Abx Consider 1 dose Toci 8 mg/kg IV (can repeat every 8 h as needed if CRS persists, max 3 doses in 24-h, max 4 doses overall) For TEC: Hold until CRS resolves	Supportive care IV fluids Infectious work up Consider Abx Toci 8 mg/kg IV (can repeat every 8 h as needed if CRS persists, max 3 doses in 24-h, max 4 doses overall) Consider dexa 10 mg IV every 12–24 h For TEC: Hold until CRS resolves	Supportive care IV fluids Infectious work up Consider Abx ICU/Pressors if needed Toci (per grade 2) If no improvement or deterioration despite 2 doses: consider other anti-cytokine agent such as anakinra Dexa 10 mg IV q6–12 h. If deterioration in 24 h: increase dose to 20 mg IV q6–12 h If deterioration in 24 h: switch to MP 2 mg/kg followed by 2 mg/kg divided 4 times per day x2–3 days, (taper as clinically indicated) For TEC: Hold until CRS resolves If recurrent grade 3 or grade 3 CRS ≥ 48 h: permanently discontinue.	Supportive care IV fluids Infectious work up Consider Abx ICU/Pressors if needed Individualized management Toci (per grade 2) If no improvement or deterioration despite 2 doses: consider other anti-cytokine agent such as anakinra Dexa 20 mg IV q6 h. If deterioration in 24 h, switch to MP 1–2 g, repeat q24-h if needed (taper as clinically indicated) or consider other anti-T cell therapies (anti-TNF, ATG, Cy) For TEC: Permanently discontinue.	

Abbreviations: Abx, antibiotics; BiPAP, bilevel positive airway pressure, CPAP, continuous positive airway pressure; CRS, cytokine release syndrome; dexa, dexamethasone; h, hours; HFNC, high flow nasal canula; ICU, intensive care unit; IV, intravenous; max, maximum; MP, methylprednisolone; q, every; TEC, teclistamab; Toci, tocilizumab.

**Table 2:** ICANS Grading and Management per the American Society of Blood and Marrow Transplantation Consensus.

	Grade 1	Grade 2	Grade 3	Grade 4	Grade 5
Presentation	ICE score 7–9	ICE score 3–6	ICE score 0–2 (If ICE 0 but pt arousable and able to perform assessment)	ICE score 0 (pt arousable and unable to perform assessment)	Death
	or	or	or		
	depressed level of consciousness but awakens spontaneously	depressed level of consciousness but awakens to voice	depressed level of consciousness: only awakens to tactile stimulus or seizures (focal, generalized, non-convulsive seizures on EEG) or raised ICP with focal/local on imaging	depressed level of consciousness: unarousable or arousable only with vigorous or repetitive tactile stimuli; stupor, coma Seizure: prolonged (>5 min) or repetitive clinical or subclinical seizures with no return to baseline in between Motor dysfunction: such as hemiparesis or paraparesis Diffuse CE on imaging and/or sxs of raised ICP: decerebrate or decorticate posturing, cranial nerve palsy, papilledema, Cushing’s triad and other	
Management	Consider dexa 10 mg PO/IV q12–24 h, de-escalated as quicky as tolerated AEDs for seizure prophylaxis For TEC: Hold until ICANS resolves	Dexa 10 mg IV every 12 h for 2–3 days, or longer if needed (taper as clinically indicated) If no improvement or worsening after 24 h: increase dexa to a max of 20 mg IV q6 h Consider non-sedating AEDs for seizure prophylaxis For TEC: Hold until ICANS resolves Hospitalization for 48 h following the next dose	Dexa 10–20 mg IV q6–12 h. If no improvement or worsening after 24 h: switch to MP 2 mg/kg followed by 2 mg/kg divided into 4 times a day; then taper Consider brain imaging If CE seen or suspected: neurologic evaluation, hyperventilation, hyperosmolar therapies (hypertonic saline, mannitol), HD-MP 1–2 g, repeat q24 h if needed (then taper) and maybe lymphotoxic agents (CY). Start non-sedating AEDs For TEC: Per grade 2	Dexa 20 mg IV q6 h. If no improvement or worsening after 24 h: switch to HD-MP 1–2 g, repeated every 24 h if needed (taper as clinically indicated). If CE seen or suspected: (per grade 3) Start non-sedating AEDs ICU level of care with mechanical ventilation if needed For TEC: Permanently discontinue	N/A

Abbreviations: AEDs, antiepileptic drugs; CE, cerebral edema; CY, cyclophosphamide; dexa, dexamethasone; EEG, electroencephalogram; HD, high dose; h, hours; ICP, intracranial pressure; ICU, intensive care unit; IV, intravenously; max, maximum; MP, methylprednisolone; PO, orally; q, every.

**Table 3:** Toxicity rates including CRS, neurotoxicity, infections and hematotoxicity in major BsAbs trials.

BsAb-Regimen	Target	Trial Phase	N	CRS, % (G ≥ 3, %)	NT, % (G ≥ 3, %)	Infections, % (G ≥ 3, %)	Neutropenia, % (G ≥ 3, %)	Thrombocytopenia, % (G ≥ 3, %)	Anemia % (G ≥ 3, %)	Ref
Teclistamab NCT03145181 NCT04557098	BCMA	I/II	165	72 (0.6)	14.5 (1.2)	76 (45)	71 (64)	40 (21)	52 (37)	[5,42]
TEC-dara NCT04108195	BCMA	I	46	61 (0)	2.1 (0)	63 (28)	54 (50)	33 (28)	46 (28)	[74]
TEC-DRd NCT04722146	BCMA	I	32	81 (0)	0%	75 (28)	75 (69)	NR	NR	[75]
Talquetamab NCT03399799	GPRC5D	II	232	D1: 77 (3) D2: 80 (0)	D1: 10 (0) D2: 5 (0)	D1: 47 (7) D2: 34 (7)	D1: 67 (60) D2: 36 (32)	D1: 37 (23) D2: 23 (11)	D1: 60 (30) D2: 43 (23)	[44]
TALQ-dara NCT04108195	GPRC5D	I	23	35 (0)	9 (4.5)	35 (17)	39 (30)	39 (22)	35 (22)	[76]
Elranatamab NCT04649359	BCMA	II	123	58 (0)	PN: 17 (1) ICANS: 3 (0)	62 (32)	43. (43)	27 (20)	45.5 (33)	[78]
Elranatamab-dara NCT05020236	BCMA	III	28	50 (0)	0 (0)	NR	29 (28)	NR	NR	[77]
Linvoseltamab (REGN5458) NCT03761108	BCMA	I	167	48 (0.6)	NR	54 (29)	29 (27.5)	21 (16)	36.5 (30)	[80,86]
ABBV-383 NCT03933735	BCMA	I	124	69 (4)	11 (0)	41 (25)	37 (34)	23 (12)	29 (16)	[81,87]
Cevostamab NCT03275103	FcRH5	I	160	80 (1)	41 (4)	42.5 (19)	18 (16)	NR	32 (22)	[90]
Alnuctamab NCT03486067	BCMA	I	47	53 (0)	NR	57 (30)	34 (30)	NR	34 (17)	[83,89,91]
Pacanalotamab NCT02514239	BCMA	I	42	38 (2)	5 (5)	33 (24)	NR	NR	NR	[84]
Pavurutamab NCT03287908	BCMA	I	75	61 (8)	8 (0)	NR (17)	23 (NR)	20 (1)	43 (NR)	[85]

Abbreviations: D1, 405-μg; D2, 800-μg; dara, daratumumab; DRd, daratumumab-lenalidomide-dexamethasone; G, grade; N, number of patients; NR, not reported; NT, neurotoxicity; PN, peripheral neuropathy; TALQ, Talquetamab; TEC, teclistamab; ref, references.
